# Impact of ceftriaxone and temocillin on fecal abundance of extended-spectrum β-lactamase producing *Escherichia coli* in a mouse model

**DOI:** 10.1371/journal.pone.0248177

**Published:** 2021-03-10

**Authors:** Rachel Chenouard, Rafael Mahieu, David Luque Paz, Estelle Marion, Matthieu Eveillard, Vincent Dubée

**Affiliations:** 1 Microbiology Laboratory, Angers University Hospital, Angers, France; 2 Center for Research in Cancerology and Immunology Nantes-Angers, UMRS1232, Institut National de la Santé et de la Recherche Médicale, Université de Nantes, Université d’Angers, Angers, France; 3 Infectious Diseases Department, Angers University Hospital, Angers, France; University of Georgia, UNITED STATES

## Abstract

**Background:**

Gut colonization by ESBL-producing *Enterobacteriaceae* (ESBL-PE) is widespread and is promoted by antibiotic exposure. Higher fecal abundance of ESBL-PE promotes the dissemination of the bacteria in the environment and is associated with increased risk of infection. Ceftriaxone and temocillin are commonly used antibiotics with a different activity on gut flora. Their impact on fecal abundance of ESBL-producing *Enterobacteriaceae* has not been studied. The objective of this study was to compare the propensity of ceftriaxone and temocillin to modify the abundance of ESBL-producing *Escherichia coli* in feces of colonized mice.

**Methods:**

Mice received broad-spectrum antibiotics in order to disrupt their normal gut flora. A CTX-M-type ESBL-producing *E*. *coli* clinical isolate was then administered orally, leading to durable colonization. Thirty days later, mice received either temocillin or ceftriaxone with drinking water at a concentration simulating human intestinal exposure. Third-generation-cephalosporin resistant (3GCR) *E*. *coli* were enumerated in feces on selective medium before, 2 days and 10 days after the end of antibiotic exposure. The experiment was performed with two *E*. *coli* isolates with different temocillin minimum inhibitory concentrations.

**Results:**

Exposure to ceftriaxone induced an increase in the fecal abundance of 3GCR *E*. *coli*. In contrast, temocillin had no effect or transiently decreased the number of 3GCR *E*. *coli*. Results obtained with the two strains were similar.

**Conclusion:**

Contrary to ceftriaxone, temocillin does not promote expansion of ESBL-producing *E*. *coli* in feces of colonized mice. Thus temocillin may be a therapeutic of choice when a temocillin-susceptible strain infection is suspected or proven to prevent the expansion of ESBL-PE in a previously colonized patient.

## Introduction

The global prevalence of extended-spectrum β-lactamase-producing *Enterobacteriaceae* (ESBL-PE) gut colonization is estimated to have increased by 5% every year between 1990 and 2015 [1]. Colonization is the first step leading to ESBL-PE infection, which carries a higher risk of treatment failure and mortality than infections due to non-ESBL producing bacteria [2,3]. The relative abundance (RA) of ESBL-PE is a predictor of ESBL infection [4], and could be positively correlated with the risk of dissemination to the environment [5]. One of the most important factors associated with a high ESBL-PE RA is a recent antibiotic exposure [6]. Few studies have evaluated the differential effect of various antibiotic exposure on the RA of ESBL-PE [7]. Antibiotics with an activity against anaerobes could have a greater effect on the expansion of antibiotic-resistant enterococci or Gram-negative bacteria [5]. In parallel, the intrinsic activity of the antibiotic may also influence the RA of ESBL-PE in feces provided that their intestinal concentration exceeds their minimum inhibitory concentrations (MIC) against these bacteria. Third-generation cephalosporins (3GC) such as ceftriaxone are recommended as first-line treatment for numerous infections such as severe pneumonia and urinary tract infections [8,9]. Their activity against anaerobes may promote the emergence of ESBL-PE in the gut of treated patients. On the contrary, temocillin is a ticarcillin analog with increased stability toward ESBL but devoid of activity against anaerobes and Gram-positive bacteria. It is marketed in Europe where it is frequently used for treatment of ESBL-PE complicated urinary tract infections [10]. This particular antimicrobial spectrum may confer to temocillin a low propensity to select ESBL-PE in colonized patients. Here, we used an animal model to compare the propensity of ceftriaxone and temocillin to modify the RA of ESBL-producing *Escherichia coli* in the feces of colonized mice. Mice received the antibiotics orally at a concentration reproducing the one observed in treated humans.

## Materials and methods

### Microbiological assay to determine human and murine fecal concentration of ceftriaxone and temocillin

To reproduce in our murine model the conditions leading to expansion of antibiotic-resistant bacteria within the intestinal microbiota of patients treated with antibiotics, we determined the concentration of antibiotics in feces of treated patients by using the microbiological method described below [11]. With this experiment, we determined the optimal ceftriaxone and temocillin concentrations to be used in our mouse model to mimic concentrations in human feces in mice feces. The same assay was performed on human and murine fecal samples. For mice, feces were collected after 2, 3, and 4 days of antibiotic administration in drinking water. Fresh feces were weighed, resuspended in a saline solution, and centrifuged at 10,000 g for 10 min. The supernatant was filter sterilized using 4 mm disposable filter units with a cut-off of 0.2 μm (Millipore). Twenty, 10 and 5 μL of the supernatant were loaded onto paper disks which were deposited on Mueller-Hinton agar plates inoculated with testing strains highly susceptible to the antibiotics dosed. The testing strains were clinical isolates of *Moraxella catarrhalis* for temocillin and *E*. *coli* for ceftriaxone, with antibiotic MICs of 0.03 mg/L. Disks loaded with known amounts of temocillin or ceftriaxone were deposited on the same plates. Plates were incubated at 37°C for 24 hours and the concentration of each antibiotic in the supernatant was deduced from the inhibition zone diameter around the paper disks [12].

### Colonization procedure

**Fig 1:**
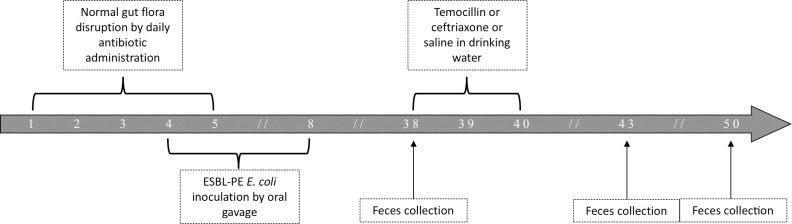
Trial profile. After gut flora disruption, mice are inoculated with the strain of 3GCR *E*. *coli* and left untreated for 30 days before exposure to temocillin, ceftriaxone or saline. Numbers represent days.

In a preceding study [13], we developed a model of stabilized gut colonization with ESBL-producing *E*. *coli* in six-week old female Swiss mice (Janvier Labs). Briefly, the normal flora was disrupted by oral administration of antibiotics and then, mice received a high inoculum of the strain of interest by oral gavage as described below. A period of thirty days was considered mandatory to stabilize the level of ESBL-producing *E*. *coli* as observed in human and in previous model [14]. This model was used in the present study with two *E*. *coli* clinical isolates: one isolate with a temocillin MIC of 1 mg/L (TEMO-S) and one isolate with a temocillin MIC of 16 mg/L (TEMO-R). We used clinical isolates from our lab collection instead of reference isolates to be able to more easily choose a temocillin-susceptible and a temocillin-resistant *E*. *coli*. As currently recommended in animal experimentation, we aimed limiting the number of mice as much as possible. Therefore, we used 33 mice (5 mice per experimental group in the temocillin resistant group and 6 in the temocillin susceptible group because of unexpected death in the first experimentation). A trial profile is provided in Fig 1. Normal gut flora was disrupted following administration of ceftriaxone (25 mg/kg/day), metronidazole (25 mg/kg/day) and vancomycin (50 mg/kg/day) by oral gavage once daily from day 1 to day 5 (the morning) [13]. Mice were randomly divided into two groups, and each group received one of the two ESBL-producing *E*. *coli* clinical isolates (5 x 10e9 CFU/mL) by oral gavage once daily on days 4, 5 and 8 (the afternoon). Thereafter, mice were left untreated for thirty days. Colonization by 3GCR *E*. *coli* was assessed regularly by culture of serial dilutions of feces resuspended in saline on a commercial selective chromogenic medium for isolation of 3GCR Gram-negative bacilli and species identification (CHROMID ESBL, bioMérieux, France). Additionally, total abundance of *E*. *coli* was evaluated by culture on a chromogenic medium allowing species identification (Brilliance UTI, Oxoid, UK). Plates were incubated for 48 h at 37°C under aerobic atmosphere.

Thirty days after the end of the colonization procedure, mice were randomly divided into three groups of five mice. One group received temocillin and the other group received ceftriaxone for three days in drinking water. The remaining mice were left unexposed to antibiotic (control group with saline). During this period, drinking water with or without antibiotics was changed every day. As mice drank this water for their usual need, we used tap water and not sterile water. The mice included in the different groups had similar characteristics (Swiss type, 6-week old, 25±3g) and had been provided by the same company (Janvier Labs, feeding by industrial granules). Fresh feces were collected (the morning) two and ten days following the end of antibiotic exposure to enumerate 3GCR *E*. *coli* as previously described. Abundance of 3GCR *E*. *coli* was expressed as CFU/g of feces. The lower limit of detection was dependent on feces weight.

The protocol was ethically reviewed and approved by the Ethical Committee in Animal Testing, Pays de la Loire, France (AFAPIS 11747-2017101111159930v2). Mice were bred in cage by four or five. All animals in the same cage received the same antibiotic.

### Statistical analysis

Results of bacterial counts are given as medians. Variations in bacterial counts are expressed as Δlog_10_ CFU/g of feces. Whenever no 3GCR bacteria could be detected, the lower limit of detection was used for all calculations. Statistical paired differences between treatment groups were assessed by 2-way ANOVA for repeated measures. We used the False Discovery Rate method to take into account the multiple comparisons.

## Results

### Determination of optimal ceftriaxone and temocillin concentration in mice drinking water

Temocillin concentrations in feces of three patients treated for complicated urinary tract infection were 0.25, 0.7, and 1 mg/g of feces 3, 3, and 5 days after antibiotic initiation, respectively. It has been shown previously that mean concentration of ceftriaxone in feces of treated patients ranges between 0.15 and 0.26 mg/g [15].

Using the same microbiological method, we assessed the proportion of each antibiotic excreted in feces after oral ingestion in drinking water (bottles). Fecal concentration of the active form of both antibiotics was approximately 70% (median result of 20 measurements) of the concentration ingested with drinking water. This proportion remained stable over time (no difference between day 2, 3, and 4), with limited interindividual variations (interquartile range of the proportion 60–120%). Therefore, mice received ceftriaxone and temocillin in drinking water at a concentration of 250 and 650 mg/L, respectively, in subsequent experiments.

### Impact of ceftriaxone and temocillin on the abundance of ESBL-producing *E*. *coli* in feces of colonized mice

**Fig 2:**
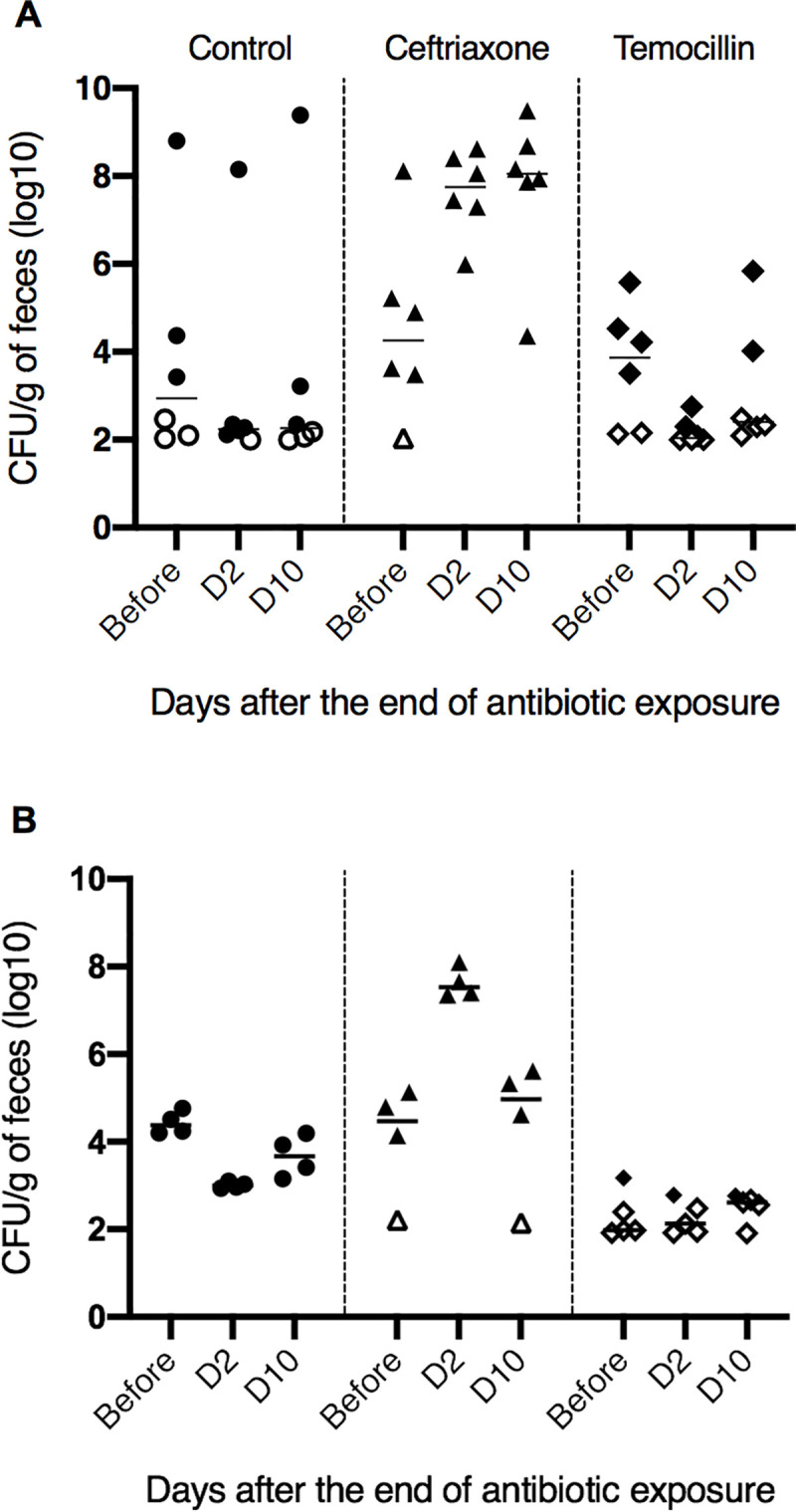
Effect of ceftriaxone and temocillin on the fecal abundance of 3GCR *E*. *coli* in a mouse model. Mice were colonized either with a temocillin-susceptible (A) or temocillin-resistant (B) CTX-M type ESBL-producing *E*. *coli*. Black horizontal bars represent medians of bacterial counts. Empty symbols represent the lower limit of detection in mice without detectable 3GCR *E*. *coli*.

During the first thirty days, the fecal abundance of 3GC-resistant (3GCR) bacteria decreased, and then stabilized at a low level similar to the level observed in colonized humans. After the end of the colonization procedure, all mice colonized with TEMO-S and TEMO-R strains had detectable 3GCR *E*. *coli* in feces. For the TEMO-S strain, thirty days after the end of colonization procedure, the median density of 3GCR *E*. *coli* was 3.5 log_10_ CFU/g of feces (Fig 2A). After a three-day oral antibiotic challenge, no change in the density of 3GCR bacteria was observed in the control group (P-adjusted = 0.24 and 0.49 at day 2 and 10, respectively). In contrast, the density of 3GCR *E*. *coli* in feces of mice exposed to ceftriaxone increased by a median of 3.5 and 4.6 log_10_ CFU/g at day two and ten, respectively, in comparison with bacterial density observed at baseline (P-adjusted = 0.03 and 0.03). Exposure to temocillin led to a non-significant decrease in the density of 3GCR *E*. *coli* bacteria at day 2, in comparison to baseline (median Δlog_10_ = -1.50 log_10_ CFU/g, P-adjusted = 0.12). At day ten, no difference was observed in comparison with baseline (median Δlog_10_ = 0.51 log_10_ CFU/g, P = 0.55). Ceftriaxone increased the number of 3GCR *E*. *coli* in feces of exposed mice significantly more than temocillin at day 2 (P-adjusted < 0.001 and < 0.01 for day 2 and day 10, respectively).

The colonization procedure with TEMO-R led to a non-different fecal abundance of 3GCR *E*. *coli* to that observed with TEMO-S. One mouse in the ceftriaxone and one in the control group died during the colonization procedure. No difference in the number of 3GCR bacteria was observed in mice exposed to temocillin at two and ten days (Fig 2B). In contrast, ceftriaxone significantly increased the abundance of 3GCR *E*. *coli* at day two (median increase 2.97 log_10_ CFU/g of feces, P-adjusted < 0.01). No significant difference was observed at day ten (median variation +0.52 log_10_ CFU/g, P-adjusted = 0.08).

## Discussion and conclusion

In this study, we assessed the impact of temocillin and ceftriaxone on the fecal abundance of 3GCR *E*. *coli* in a mouse model of ESBL-producing *E*. *coli* gut colonization. For this purpose, antibiotics were given orally in order to reproduce antibiotic concentrations observed in treated humans. Our results show that ceftriaxone leads to a major increase in the excretion of 3GCR *E*. *coli* after the end of antibiotic exposure, whereas temocillin does not. This may arise from two major differences between these two antibiotics. Contrary to ceftriaxone, temocillin lacks activity against anaerobes, and is active against most ESBL-producing *E*. *coli*. A vast literature supports the hypothesis that activity against anaerobes is a major determinant of the ability of an antibiotic to promote gut colonization by antibiotic-resistant bacteria [16,17]. The impact of antibiotic intrinsic activity on excretion of resistant bacteria has been less studied. In our study, a non-significant decrease in the number of the temocillin susceptible strain of 3GCR *E*. *coli* was observed during temocillin exposure (Δlog_10_ = -1.50 log_10_ CFU/g) suggesting that temocillin may have a direct effect on the resistant bacteria. Moreover, several reports show that carbapenems, despite their activity against anaerobes, decrease the fecal density of ESBL-producing bacteria or do not promote gut colonization [7,18], suggesting that antibiotics with activity against resistant bacteria could have a favorable profile. The differences between human and mouse gut microbiota may query the conclusions of our study. However, most conclusions of animal models assessing the impact of antibiotics on selection of resistant bacteria have been confirmed in human clinical studies [19]. Another limitation is the number of mice included in the protocol, but this choice was justified by a balance between the minimum number needed to provide significative results and the respect of ethic in animal experimentation.

Third-generation cephalosporins are recommended as first-line therapy for treatment of complicated urinary tract infections [20]. Our results, in line with others, suggest that prescription of these antibiotics promotes ESBL-selection in the gut of treated patients, which is the first step to infection and may be associated with an increased risk of transmission of resistant clones. Temocillin is active against most *E*. *coli* isolates responsible for urinary tract infections [21] and our study shows that it has minimal impact on the fecal abundance of ESBL-producing *E*. *coli*. Thus, in a context of growing ESBL burden, temocillin is an interesting option for empirical therapy of complicated urinary tract infections.

## Supporting information

S1 File Temocillin susceptible *E*. *coli*. (PZFX)

S2 File Temocillin resistant *E*. *coli*. (PZFX)
